# Proposal of a New Conformal Factor and Normal Tissue Penalty Factor for Radiosurgery Treatments

**DOI:** 10.7759/cureus.44800

**Published:** 2023-09-06

**Authors:** Venkatraman Murali

**Affiliations:** 1 Medical Physics, Private Practice, Chennai, IND

**Keywords:** ntpf, conf, ci, reference isodose, normal tissue, target volume

## Abstract

The quality of a treatment plan is evaluated by the conformality of the prescribed isodose around the target and the homogeneity of dose distribution inside the target. Presently, to check the target volume conformality, a number of published conformity indices are in use. Most of these indices are based on the target volume coverage by prescribed isodose, with respect to the total volume of the target. Some take into account the normal tissue covered by the prescribed isodose and suitably weigh the target coverage to evaluate conformity. In this study, for the irradiation of normal tissue by the prescription isodose, a normal tissue penalty factor is proposed and for the target conformality, a new conformal factor is proposed by applying this normal tissue penalty factor to the target coverage. The proposed conformal factor is evaluated for a few sample analytical cases and the results are compared with those obtained using the published conformity indices.

## Introduction

The basic principle behind external beam therapy is to maximize the dose to the target and minimize the dose to the surrounding normal tissue. This is valid more so in the case of stereotactic radiosurgery (SRS), where a large dose of radiation is delivered to the target in a single fraction, and in the case of stereotactic radiotherapy (SRT)/stereotactic body radiotherapy (SBRT) where hypofractionated doses are delivered. To assess the conformity of the treated target volume (TV) by the reference isodose (RI) used for prescription, a number of formulae are available and are in use currently [1-5].

In 1993, the Radiation Therapy Oncology Group (RTOG) proposed a conformity index (CI) [1], which is a ratio of the volume of the reference isodose (V_RI_) to TV.

CI (RTOG) = V_RI_/TV …….(1)

Paddick [2] proposed a CI, in which the proportion of the target coverage by the RI, is balanced by the proportion of the V_RI_ inside the TV

CI (Paddick) = (TV_RI_ /TV) x (TV_RI_/V_RI_) ........(2)

where TV_RI_ is the volume of the target covered by RI.

In 2003, Lomax and Scheib [3], proposed a CI which is a ratio of TV_RI_ to V_RI_. This is also termed as healthy tissue CI [3]:

CI (Lomax) = TV_RI_/V_RI_ ………(3) 

As most of the published formulae to estimate the CI are aimed to assess the coverage of the target by the RI, the inclusion of the normal tissue within the RI is not explicitly expressed.

## Materials and methods

Wagner et al. published an index for the SRS plans [6]. This is an average of conformity and gradient scores and is termed as conformity/gradient index (CGI). This CGI is computed based on the TV, TV_RI_, the effective radius of the V_RI_, and the effective radius of the isodose line, which is equal to 50% of the V_RI_.

Wu et al. published a different index in 2003, namely conformity distance index (CDI) [7]. It is defined as the average distance between the TV and RI. Accordingly, a CDI value of 1 means the average distance of the RI from the TV is about 1 mm. The CDI takes into account the volume of normal tissue that receives the RI and above, the TV, the surface areas of the TV, and V_RI_.

In 2014, Park et al. proposed a distance-based conformity index, called CI_distance _[8]. In this method, equiangular lines at one-degree intervals are drawn from the centroid of the TV, three-dimensionally. The distances of these lines from the centroid to the point of intersections at the surfaces of TV and V_RI_ are measured, and the difference is calculated. The average difference is analyzed, which is the CI_distance_. The authors themselves have concluded that since CI_distance_ is an average value, it doesn’t always provide the exact information on the irradiation of normal tissue.

As has been mentioned earlier in the introduction, the amount of normal tissue included within the RI is not clearly expressed. The aim of this article is not to review the indices published so far and bring out the pros and cons associated with each CI, but to propose a factor that is read along with the estimation of normal tissue irradiation in the RI. To estimate the inclusion of the normal tissue within the RI, a normal tissue penalty factor (NTPF) is proposed in this study and is shown in equation (4). This is the ratio of the volume of normal tissue included in the RI (NT_RI_) to the V_RI_. When it is attempted to increase the TV coverage by the RI, it is mostly associated with the possibility of including more normal tissue around the TV. This scenario happens especially when the TV is close to a dose-limiting critical organ on one side. The presence of critical organs on one side of the TV impacts the dose distribution and makes it shift to the opposite side. The result is that adequate TV coverage might be achieved but at the cost of including more normal tissue. Hence, a new conformal factor (CONF) is proposed, which takes into account the NT_RI_. The CONF is evaluated by subtracting the NTPF from TC_RI_, which is the target coverage index shown in equation (5). The CONF is clearly expressed in equation (6). In other words, the actual target coverage is penalized due to the inclusion of normal tissue. The basic intention behind this proposal is to evaluate the target conformity in conjunction with normal tissue irradiation, especially in high-precision treatments like SRS and SBRT.

Normal Tissue Penalty Factor, NTPF = NT_RI_/V_RI_ ………(4)

where, NT_RI_ = V_RI_ - TV_RI_

Target Coverage Index, TC_RI_ = TV_RI_/TV ………(5)

Conformal Factor, CONF = TC_RI_ - NTPF ……….(6)

Equation (4) returns a minimum value of 0, when the NT_RI_ is 0. This happens when the RI snugly conforms to the TV and when the RI is within the TV. The maximum value would be 1, when the RI is totally out of the TV. Equation (6) returns a maximum value of 1, when V_RI_ =TV_RI_=TV, a case of perfect conformality, and a minimum value of -1 when TV_RI_ = 0, a case of complete miss of the target and irradiation of normal tissue only. As is seen clearly from equations 4-6, both NTPF and CONF are mere numbers and do not carry any units.

## Results

**Figure 1:**
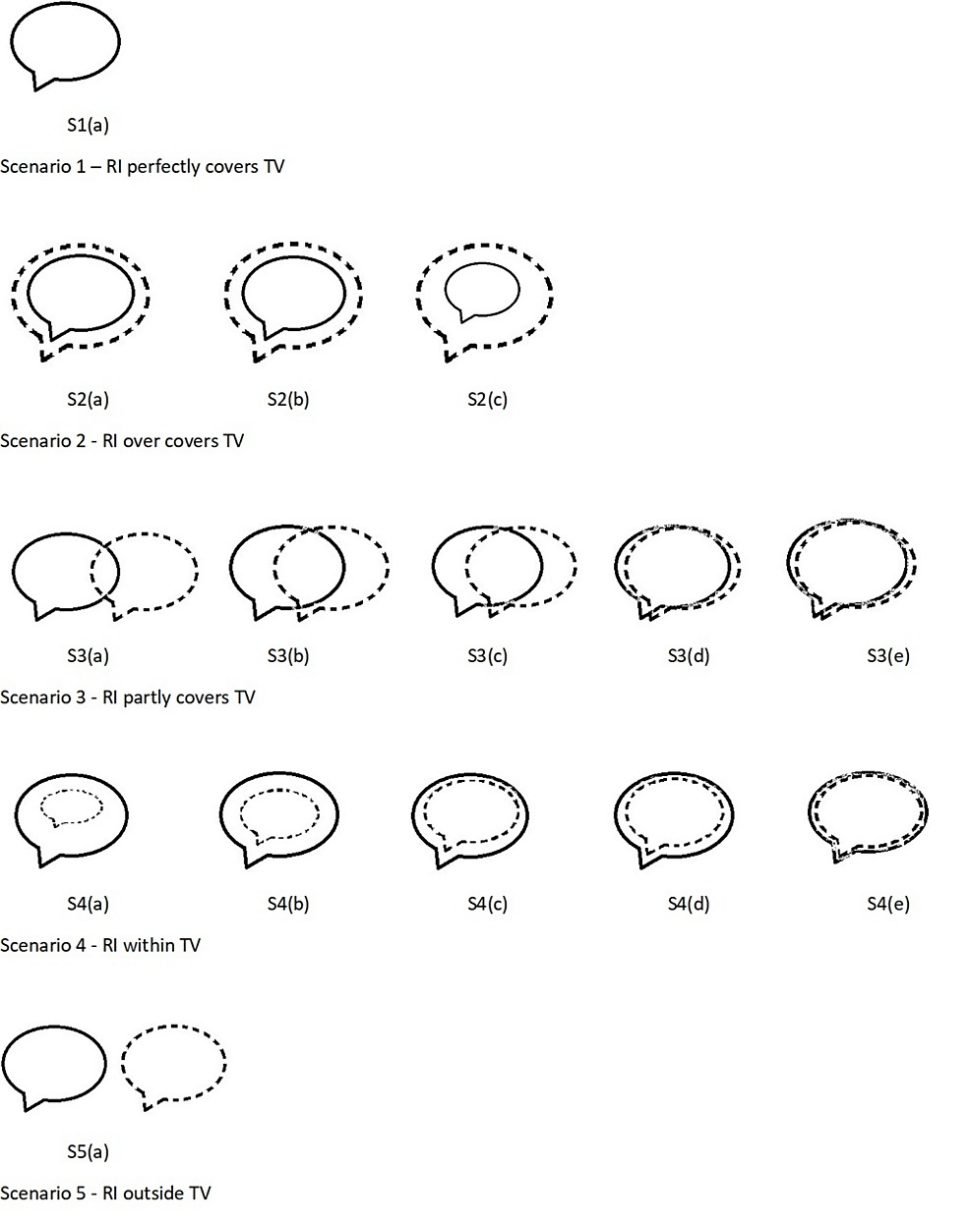
Scenarios 1-5 taken for analysis. In S1 (a), the RI conforms to TV; hence, only a solid line is seen. The figure is for representative purposes only and not drawn to scale. Solid Line: Target Volume (TV); Dotted Line: Reference Isodose (RI)

The CONF and NTPF are calculated for a few analytical cases in five different scenarios, the diagrams of which are shown in Figure 1. Since the proposal of these factors is exclusively for SRS/SRT, a TV of 5 cc is considered. To portray the scenarios considered very clearly, the TV is so chosen that neither it is regular nor highly irregular. The TV is shown with a solid line, while the RI is with a dotted line. Though some of these cases are not acceptable in clinical situations, they are considered only for the sake of study and analysis. CI (RTOG, Lomax, Paddick) values are calculated using equations (1) to (3) for these cases, and the results are presented in Table 1 for comparison. Since the diagrams shown in scenarios 1-5 could not be incorporated in Table 1, the corresponding diagram labels are mentioned in column 2 of Table 1.

**Table 1:** Calculated CI, CONF and NTPF values for scenarios 1-5 Column 1 in the table shows in percentage, the RI volume which overlaps the TV. The CI, CONF, NTPF values are mentioned up to three decimals just for comparison only; TV= 5 cc Representative Diagrams indicate the diagram labels from Figure 1. TV: target volume; CI: conformity index; CONF: conformal factor; NTPF: normal tissue penalty factor; RI: reference isodose

Volume of RI overlap- Relative to TV	Representative Diagram	TV_RI _( cc)	V_RI _( cc)	NT_RI _( cc)	CI (RTOG)	CI (Lomax)	CI (Paddick)	CONF	NTPF
Scenario 1 – RI perfectly covers TV									
100 %	S1(a)	5	5	0	1.000	1.000	1.000	1.000	0.000
Scenario 2 - RI over covers TV									
120 %	S2(a)	5	6	1	1.200	0.833	0.833	0.833	0.167
160 %	S2(b)	5	8	3	1.600	0.625	0.625	0.625	0.375
200 %	S2(c)	5	10	5	2.000	0.500	0.500	0.500	0.500
Scenario 3 - RI partly covers TV									
20 %	S3(a)	1	5	4	1.000	0.200	0.040	-0.600	0.800
50 %	S3(b)	2.5	5	2.5	1.000	0.500	0.250	0.000	0.500
80 %	S3(c)	4	5	1	1.000	0.800	0.640	0.600	0.200
90 %	S3(d)	4.5	5	0.5	1.000	0.900	0.810	0.800	0.100
95 %	S3(e)	4.75	5	0.25	1.000	0.950	0.902	0.900	0.050
Scenario 4 - RI within TV									
20 %	S4(a)	1	1	0	0.200	1.000	0.200	0.200	0.000
50 %	S4(b)	2.5	2.5	0	0.500	1.000	0.500	0.500	0.000
80 %	S4(c)	4	4	0	0.800	1.000	0.800	0.800	0.000
90 %	S4(d)	4.5	4.5	0	0.900	1.000	0.900	0.900	0.000
95 %	S4(e)	4.75	4.75	0	0.950	1.000	0.950	0.950	0.000
Scenario 5 - RI outside TV									
0 %	S5(a)	0	5	5	1.000	0.000	0.000	-1.000	1.000

## Discussion

The proposed CONF computation needs to be checked against the computed values of a few published CIs. Only then can the proposal be validated and justified. Hence CI (RTOG, Lomax, Paddick) are chosen, as these CI computations are very simple and easy. On analyzing the results, it is seen that in scenario 1, for a perfectly covered TV, the CI (RTOG, Lomax, Paddick) and the CONF values are equal to 1. As the NT_RI_ is 0, the NTPF is also 0. In scenario 2, where the RI overcovers the TV, three different cases are presented with V_RI_, 120%, 160%, and 200% of the TV. The CONF matches well with CI (Lomax, Paddick). As V_RI _increases, the NT_RI_ also increases, which in turn increases NTPF and decreases CONF. This shows that the CONF estimates the target conformity, applying the penalty for normal tissue irradiation appropriately. As the V_RI_ increases, the CI (RTOG) also increases. The reason for this is that CI(RTOG) takes into consideration only the numerical value of the V_RI,_ rather than its geometric location relative to TV.

In scenario 3, where the RI partly covers the TV, five cases are shown. There is a shift of the RI to one side of the target, keeping V_RI _= TV. The CI (RTOG) for all five cases is equal to 1, as V_RI_=TV. When the RI covers 20%, 50%, 80%, 90%, and 95% of the TV, CI (Lomax) respectively is 0.2, 0.5, 0.8, 0.9, and 0.95, which is obviously the fraction of TV coverage. CI (Paddick) for the same cases, yields lesser values compared to CI (Lomax), as TV coverage is proportionately weighted for the RI inside TV. The NTPF yields values proportionate to the NT_RI_. Since the NTPF is applied as a penalty to TC_RI_, the CONF is still less compared to the corresponding CI (Paddick). As per equation (6), the CONF will be equal to zero, when the TC_RI_=NTPF. In other words, the target conformity is nullified, if TV_RI_ is 50% of TV and NT_RI_ is 50% of V_RI_. In scenario 4, again five cases are shown, where the RI covers 20%, 50%, 80%, 90%, and 95% of the TV as in scenario 3, but the difference is that the RI is within the TV and NT_RI_ is 0. While, CI (RTOG) estimates values corresponding to the coverage of TV, the CI (Lomax) yields a value of 1 for all the cases, as TV_RI_=V_RI_. CI (Paddick) and CONF both yield similar values, corresponding to the coverage of the TV. Since NT_RI_ is zero, the NTPF is zero and hence it has nil impact on the CONF. Interestingly, it is observed that the CI (RTOG, Lomax) values are getting swapped in scenarios 3 and 4 for the same TV_RI_. In scenario 5, as the RI is outside the TV, the TV_RI_ is 0; hence, CI (Lomax, Paddick) is also 0. CI (RTOG) is 1, as this index is not a function of TV_RI_. The CONF returns a value of -1 as NTPF is 1, indicating a complete miss of the target and irradiation of normal tissue only.

In scenarios 1 and 4, the NT_RI_ is 0, and hence, NTPF has no impact on the CONF. In scenario 2, though the TV is fully covered, since the normal tissue is also irradiated to the same level or more of RI, the NTPF plays its role. As the NT_RI_ increases, so does the NTPF, which brings down the CONF for the TV, which is otherwise fully covered by the RI. The same trend is seen in scenario 3, where the TV is partly covered. As a matter of fact, the CONV turns negative when the NT_RI_ exceeds 50% of V_RI_. Of course, this depends on the V_RI_, TV_RI_, and TV. In Table 1 (line 2 of scenario 3), the case is presented with TV=V_RI_=5 cc; TV_RI_= NT_RI_=2.5 cc (50% of V_RI_), and hence, CONV is 0. Scenario 5 is only for the sake of analysis and is not clinically relevant.

It is seen that the proposed NTPF and CONF definitely would be useful in scenarios 2 and 3 to assess the conformity along with the normal tissue irradiation when planning for an irregular target. An example of this is the planning for an arteriovenous malformation (AVM). Along with a nidus drawn, healthy blood vessels also get into the RI. The proposed NTPF and CONF would enable the planning physicist to generate multiple plans, by bringing down the value of NTPF close to 0 and increasing the value of CONF towards 1. The radiation oncologist would have the advantage of choosing an appropriate isodose for prescription, based on the NTPF and CONF values. 

Limitations

In this study, only analytical data from the simulated scenarios could be presented and discussed. At the time of writing this article, there was no access to patient data. Otherwise, results of retrospective analysis would have also been included to substantiate the proposal of NTPF and CONF. This study is limited only to target dose conformity and normal tissue irradiation. Moreover, since this study is on the simulated models, the impact of reducing the NTPF on the dose homogeneity of the TV or the dose gradient outside the TV is not known. Multiple treatment plans need to be generated on the real patient data by varying the NTPF and evaluated.

## Conclusions

In high-precision radiation treatments like SRS and SBRT, as very high doses are delivered, sub-milli-meter accuracy in targeting is aimed using image guidance. During the planning process, while delineating the target on CT images, additional modalities of imaging like MR, positron emission tomography (PET), etc. are utilized. The basic idea is that the target needs to be perfectly delineated and treated without including much of the normal tissue. Similarly, while planning it needs to be aimed at covering the TV as much as possible, minimizing the irradiation of the normal tissue. Though, it is always desirable to have the RI cover only the TV and achieve perfect conformity, it may not be always possible in practice. Normal tissue does get irradiated while trying to improve the coverage. Hence, there is a need to estimate the NT_RI_ and evaluate the conformity. The CONF takes into account the NT_RI_ and estimates the conformity of the TV, as it is clearly evident from the results given for scenarios 3 and 4, for the same TV_RI_. The NTPF clearly indicates the inclusion of normal tissue as a fraction of V_RI_. This would help the treatment planning physicists to keep the NTPF tending towards 0, while trying to achieve a reasonably good coverage of the TV.

Two major points need to be mentioned here. One is that the NTPF and CONV directly convey the quality of a plan without requiring any interpretation. The other one is that the computation of NTPF and CONF doesn’t involve complex mathematical operations for the treatment-planning computers to handle. Institutions can set their own policy of NTPF and CONF to evaluate the plans. Recording of NTPF along with CONF, in the treatment plan dose statistics, would enable the radiation oncologists to compare different plans and choose a better one.
